# Public Knowledge and Attitudes Towards Clinical Trial Participation: A Mixed-Method Study in Bagamoyo District, Tanzania

**DOI:** 10.3390/ijerph23050633

**Published:** 2026-05-11

**Authors:** Stanslaus Mghanga, Alan Elias Mtenga, Liliane Pasape, Ally Olotu

**Affiliations:** 1 The Nelson Mandela African Institution of Science and Technology, Arumeru, Arusha 23318, Tanzania; a.mtenga@kcri.ac.tz (A.E.M.); liliane.pasape@nm-aist.ac.tz (L.P.); 2 Ifakara Health Institute, Bagamoyo Branch, Bagamoyo P.O. Box 74, Tanzania; aolotu@ihi.or.tz; 3 Kilimanjaro Clinical Research Institute, Longuo, Moshi 25116, Tanzania

**Keywords:** clinical trials, awareness, public knowledge, attitudes, participation, mixed methods, perception, Tanzania

## Abstract

Background: Clinical trials are important for advancing medical knowledge and improving healthcare delivery. However, participants’ knowledge and attitudes towards clinical trials remain a key challenge affecting clinical trial recruitment and participant retention. Therefore, this study aimed to assess the knowledge and attitudes of the Bagamoyo district towards participation in clinical trials. Methods: A convergent parallel mixed-methods study was conducted among adults in the Bagamoyo district. Multistage stratified random sampling was used to select participants. Quantitative data were analysed descriptively and using logistic regression, while qualitative data were analysed thematically using NVivo. Results: Among 394 recruited participants, 293 (74.4%) were female and 101 (25.6%) were male. Most participants had a primary-level education (266, 67.5%), while 128 (32.5%) had secondary or tertiary education. The majority were married (297, 75.4%) and 97 (24.6%) were either separated or unmarried. Regarding economic status, 244 participants (61.9%) earned less than TZS 50,000. General knowledge of clinical trials was low, with most participants scoring below 60%. However, we found a positive attitude towards participation in clinical trials. Logistic regression revealed that poor knowledge was significantly associated with being male (AOR, 22.95 (95% CI: 10.27–51.28, *p* = 0.001)), age above 55 years (AOR of 2.43 (95% CI: 1.29–4.55, *p* = 0.006)), and unemployment (AOR of 2.39 (95% CI: 1.27–4.53, *p* = 0.007)). Positive attitudes towards clinical trial participation were significantly associated with being female (AOR) 7.61 (95% CI: 4.32–13.39, *p* < 0.001), age 44 years and below, (AOR: 2.22 (95% CI: 1.27–3.86, *p* = 0.005), and employment (AOR of 1.89 (95% CI: 1.08–3.32, *p* = 0.03). Conclusions: Despite low levels of knowledge, the general population in the Bagamoyo district demonstrated a high willingness to participate in clinical trials. To address the knowledge gap, targeted educational interventions should focus on older adults and the unemployed. Furthermore, policies supporting community outreach and awareness campaigns may help strengthen public understanding and sustain positive attitudes toward clinical research.

## 1. Introduction

Clinical trials are investigations that assign one or more participants to one or more interventions (either a control or a placebo) to evaluate the effects of interventions on behavioural or biological outcomes relevant to health [1]. They involve testing new drugs, treatments, or interventions related to human subjects to determine their safety, effectiveness, and important side effects [2,3]. Clinical trials are an essential part of the drug development process and play a vital role in improving the health of people around the world [2,4]. In addition, clinical trial information supports healthcare professionals and patients in understanding the benefits and risks of the tested interventions [2]. Finally, clinical trials offer a forum for researchers to communicate their discoveries about various disease therapies [5].

Because clinical trials are meticulously planned to reduce bias and provide strong evidence, they are regarded as the gold standard for assessing the safety and efficacy of experimental medical treatments [6]. Clinical trials are conducted by following a well-defined protocol and are usually performed in multiple phases, starting with small-scale Phase 1 trials, and then Phase 2 and Phase 3 and then Phase 4. The latter involves a large cohort if the results are promising [2]. For clinical trials to provide reliable results and address the research question at hand, sufficient sample sizes and high retention of human participants are required [7,8]. Therefore, to increase community participation in research, it is necessary to develop such interventions with a thorough understanding of the factors influencing recruitment and sustained participation within our community, as well as to improve the relationship between researchers and study participants [9,10].

Despite the benefits of clinical trials, recruitment and retention of participants in clinical trials have been reported as major challenges [11,12,13,14]. One study reported that 85% of all clinical trials fail to meet recruitment goals [15], while another reported that fewer than 31% of trials achieve their original recruitment goal and 53% request extensions [16]. Inadequate recruitment also increases the cost of research in terms of time, resources, and personnel. It may delay the completion of drug development processes and the approval of new interventions [17,18]. This could all be due to negative attitudes towards clinical trials. The literature shows that a lack of awareness, misinformation, negative perceptions, and fear or mistrust of the clinical research process are the leading causes of poor participation and poor retention of participants in clinical trials [19,20,21,22]. The evidence of more than 37% of investigators in India shows that inadequate sample size in CTs is due to poor knowledge among participants, and more than 63% investigators agreed that enhancing positive awareness among participants will increase the participation in CTs [14]. Furthermore, a positive attitude in the community is very important for participation in CTs [23].

Tanzania is among the countries reported to have limited knowledge, attitudes, and negative perceptions among participants in different health programmes. A study performed in three other districts of Tanzania assessing knowledge, attitudes and practices (KAP) regarding antimicrobial use (AMU) and antimicrobial resistance (AMR) among communities found that the community has a moderate level of knowledge, attitude and practice, which was significantly associated with participant age and level of education [24]. Other studies were conducted to assess the knowledge, attitude, and practice towards cervical cancer control among university students. They found that more than 75% had poor knowledge and 82% had negative attitudes towards cervical cancer [25,26].

According to the African Academy of Sciences (AAS) clinical trial community, only 2% of all vaccine clinical trials take place in Africa [27]. This indicates a high magnitude in the knowledge gap towards clinical trials, which is why the dissemination of information on clinical trials needs to be given close attention, especially fromf a Tanzanian perspective. However, no studies have been conducted in Tanzania to assess the knowledge levels and attitudes of the general community towards participation in clinical trials.

This study aimed to assess the level of knowledge and attitudes of the population of the Bagamoyo district towards participation in clinical trials. The findings of this study will identify misconceptions, knowledge gaps, and underlying factors that may hinder clinical trial participation. It will guide investigators and clinical research practitioners in directing an intervention to improve the general perception of research among community members and in developing novel recruitment and retention strategies to improve clinical trial outcomes. Considering that Bagamoyo District hosts a potential clinical trial centre in Tanzania (Ifakara Health Institute Clinical Trial), where various malaria vaccine trials are being conducted, the study will provide insights to strengthen recruitment efforts, raise awareness, and promote greater community participation in clinical trials.

## 2. Materials and Methods

### 2.1. Study Design and Setting

A convergent parallel mixed-methods study was conducted among the general community of Bagamoyo district. A convergent mixed-methods design allows the collection and analysis of both quantitative and qualitative data simultaneously, thus providing both breadth (via survey data) and depth (via interviews and focus groups) in understanding community perceptions of clinical trials. The data are analysed separately, and the results are merged or integrated to compare, contrast, or combine the findings [28]. We employed this mixed-methods approach to robustly identify patterns in knowledge and attitudes towards clinical trial participation and to explore possible underlying reasons or perceptions from the community perspective, complementing the quantitative findings. Bagamoyo was chosen because of its clinical trial facility, where we believed our data could be valid and reliable for answering our research questions.

### 2.2. Sample Size Estimation and Sampling Method

The sample size was calculated using a significance level of 0.05, a confidence interval of 95%, a proportion of 50% (used as a conservative estimate to ensure sufficient sample), a Z-score of 1.96, and a design effect (stratified sampling) of 1.5.

$$n=\frac{z^{2}*p\left(1-p\right)}{e^{2}}\;\;n=\frac{1.96^{2}*0.5\left(1-0.5\right)}{0.05^{2}}=384$$

where

n—sample size;

z—z-score;

e—margin of error;

p—expected proportion.

The study population consisted of households residing in Bagamoyo district. A multistage stratified random sampling method was used. In the first stage, Bagamoyo district wards were divided into two strata based on their prior inclusion in clinical trial activities (previously included vs. not included). This information was obtained through a desk review of records from the IHI Bagamoyo Clinical Trial Facility. In the second stage, two villages were randomly selected from each stratum, resulting in a total of four study villages. Within each village, systematic random sampling was used to select households.

### 2.3. Inclusion and Exclusion Criteria

Participants had to be 18 years of age or older, living in the Bagamoyo district, and able to provide written informed consent. The visitors and tourists in the study areas were excluded.

### 2.4. Conceptual Framework

**Figure 1:**
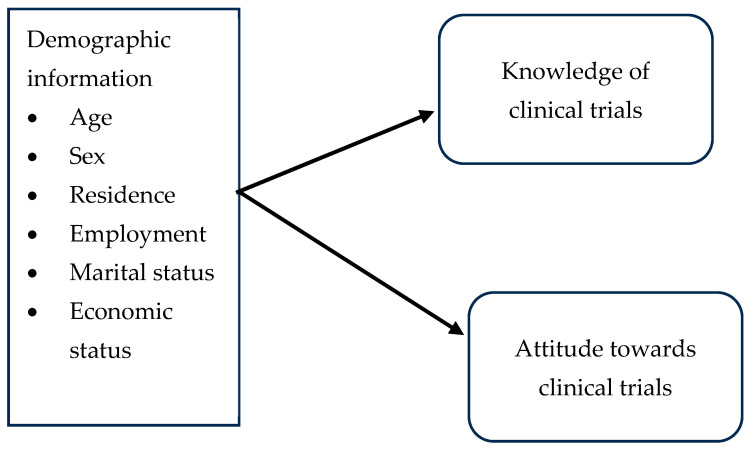
Conceptual framework to assess knowledge of and attitude towards clinical trials.

This study used the KAP (knowledge, attitude, and practice) survey model; however, the result of the current study only reports the knowledge and attitude items. The KAP survey has been used in various studies of health-seeking behaviour. It has proven to be one of the best instruments for assessing knowledge, attitudes, and practices about health related to social, traditional factors and idea that each person has of diseases [29]. Today, KAP surveys have become the most commonly used studies to demonstrate societal context within public health research [30,31,32]. These surveys are easy to set up, can give accurate results, are easy to interpret, and are relevant to a particular context. For more detailed information, see Figure 1, below.

### 2.5. Ethical Aspects

The study commenced after receiving approval from the Ifakara Health Institute-Institutional Review Board (IHI-IRB), by decision number IHI/IRB/NO: 41-2023. On 20 February, the data collection started, and ended on 31 May 2024. The study was carried out in accordance with the ethical standards set by the relevant institutions or national research regulators, and with the principles of the Declaration of Helsinki.

### 2.6. Data Collection

Quantitative data: The KAP (knowledge, attitude, and practice) survey questionnaire was used to collect the data. However, this study reports only on findings related to knowledge and attitude. The questionnaire was prepared in English, translated into Swahili, and administered via the Open Data Kit (ODK) application integrated with the Kobo Toolbox (https://www.kobotoolbox.org/, accessed on 4 October 2025). Responses were recorded on a 3-point Likert scale: “agree”, “disagree”, or “not sure”.

Qualitative data: Simultaneously with the quantitative data collection, focus group discussions and in-depth interviews were conducted. Ten participants were purposively selected and invited to the in-depth interviews, including one community healthcare worker. In addition, we conducted 4 group discussions (FGDs) with 8 participants each. The FGD participants included community leaders, religious leaders, influential individuals, community members who had previously participated in clinical trials, and community members who had never participated in clinical trials. This number was sufficient to reach saturation. Saturation was defined as “having no new emerging themes related to clinical trial participation” during the interview. The interviews were conducted at mutually agreed locations and times by the trained research assistants. All interviews were conducted in Swahili, which is the common language among the participants.

### 2.7. The Data Analysis

The categorisation of the knowledge score was calculated by assigning 1 point for each correct score “yes” and zero in the case of a “no” or “do not know” response. A “No/don’t know” score was combined because they all do not represent a positive remark. The total knowledge score was calculated by summing the scores for each participant, with a maximum obtainable score for each participant. The sum score was converted into a percentage knowledge score. For ease of comparison, the knowledge status was divided into ‘low’ and ‘high’ knowledge based on mean knowledge score. Respondents, who scored below the mean knowledge score, were categorised as having “low knowledge”. In contrast, respondents who had equal or greater than the mean knowledge score were categorised as having “high knowledge” regarding clinical trials.

The categorisation of attitude scores was based on assigning 1 point for each correct or favourable response (e.g., “Agree” to positively framed statements), while “Disagree” or “Not Sure” responses were scored as zero because they do not represent a positive statement. Total attitude scores were calculated as continuous variables by adding the respondent’s number of appropriate answers to obtain the total sum score. The total sum score was then converted to percentages. The attitude status was also dichotomised as positive or negative, with the mean score used as the cut-off point for group comparison. Respondents who scored below the mean were categorised as having a negative attitude, while respondents who had a score equal or greater than the mean score were categorised as having a positive attitude.

SPSS version 17.0 was used to analyse the data. Descriptive analysis was performed and presented as frequency and percentage. The continuous variables were presented as mean and standard deviation (mean ± SD). The sample characteristics were reported using descriptive statistics. The data were further analysed descriptively to achieve an overview of knowledge and attitude frequencies. The data were tested for normality using the skewness coefficient, which indicated normality. Univariate analysis was performed through chi-square tests and binary logistic regression to examine the degree of correlation between the primary outcome variables of interest (knowledge and attitude) and the independent variables (age, gender, educational level, marital status, employment status and income), because the outcome variables were dichotomous (higher knowledge vs. low knowledge; positive attitude vs. negative attitude). To identify characteristics that significantly influence the possibility of having higher knowledge or a positive attitude while adjusting for potential confounders, this method is suitable for modelling the probability of a binary outcome as a function of many predictor variables [33].

The audio recordings from the focus group discussion and in-depth interviews were loaded into a computer for transcription and translation. The transcript was translated into an English version by an experienced researcher (S.M. and A.E.M.). An initial codebook was developed by the corresponding author (S.M.) and then shared with other authors for agreement. The transcript and codebook were loaded into NVivo 14 for coding and data organisation. The data was analysed inductively by reading and re-reading the transcript to identify the emerging themes.

## 3. Results

### 3.1. Participants’ Demographic Attributes

Data were obtained from 394 participants, including 293 women (74.4%) and 101 men (25.6%). The largest proportion of participants, 140 (35.5%), were aged 18–44 years. Most respondents had attained only primary education or no education (266, 67.5%), and the majority were married (297, 75.4%). Regarding income, 244 participants (61.9%) reported earning less than 50,000 Tanzanian shillings per month (see Table 1).

A total of ten in-depth interviews were conducted involving participants with different backgrounds, including one community healthcare worker, two (2) religious leaders, two (2) village leaders, two (2) participants who had previously participated in a clinical trial, one (1) participant who is currently participating in a CT, and two (2) who had never participated in clinical trials.

### 3.2. Knowledge of Clinical Trials in the General Population of Bagamoyo District

The knowledge responses revealed that respondents had moderate knowledge in certain areas, such as understanding that participation in a clinical trial is voluntary (67.5%), that the medicines are tested before they are used in humans (60.3%), and that participants may withdraw from the CTs at any moment (60.2%). Low knowledge was mainly evident in domains such as the meaning of clinical trials (47.2%), the difference between clinical trials and standard care (46%), and the risks involved in participating in clinical trials (25.9%). The lowest levels of knowledge were observed in key methodological and ethical areas. Only 8.4% of respondents were familiar with the concept of randomisation, 5% understood the use of placebos, and 14% were aware of the role of ethics review committees in overseeing clinical trial conduct, as can be seen in Table 2.

A logistic regression analysis was conducted to identify the association of participants’ characteristics with knowledge of clinical trials. The results revealed that sex was significantly associated with knowledge levels, with women being more likely to have higher knowledge compared to men (AOR = 18.1; 95% CI: 8.7–37.9; *p* = 0.001).

Younger age was also significantly associated with knowledge, with individuals aged 18–44 years more likely to have more knowledge than those aged 45+ (AOR = 3.1; 95% CI: 1.7–5.7; *p* = 0.001). Furthermore, participants with low social economic status of less than TZS 50,000 were more likely to have greater knowledge than participants with higher social economic status (AOR = 2.2; 95%CI: 1.3–3.6) and unemployed or self-employed participants were more likely to have greater knowledge than those who were employed or were housewives (AOR = 2.39; 95% CI: 1.2–4.2; *p* = 0.01). Detailed regression results are presented in Table 3.

### 3.3. Attitudes Toward Participation in the CTs Among the General Population in Bagamayo District

Overall, the study population demonstrated a mixed or bidirectional response pattern regarding their attitude toward clinical trials, with 89.6% expressing a willingness to participate in a CT if adequate information was provided, 80.7% reporting that they would recommend others to participate in clinical trials, and 90.9% recognising the benefits of clinical research. At the same time, a startlingly high percentage (53.8%) concurred that clinical trials harm society, and 31.5% believed that study subjects are treated like guinea pigs.

In addition, moderate levels of concern were observed regarding privacy protection (75.1%), trust in the information provided (70.6%), and the expectation of financial compensation (66.2%) during participation, as seen in Table 4.

After adding age, gender, education level, socioeconomic status, and marital status to the logistic regression model, the result showed that positive attitudes were significantly associated with being female (AOR = 6.2; 95% CI: 3.6–10.8; *p* < 0.001); younger age (AOR = 2.2; 95% CI: 1.2–3.9; *p* = 0.007); employment status, with employed individuals more likely to report positive attitudes compared to those who are unemployed (AOR = 2.3; 95% CI: 1.3–4.1; *p* = 0.004); and low social income (AOR = 2.1; 95% CI: 1.3–3.4; *p* = 0.003). For more detailed results, refer to Table 5.

### 3.4. Qualitative Findings

#### 3.4.1. Knowledge of Clinical Trials

During our in-depth interviews and focus group discussions, we discovered various perspectives regarding knowledge of clinical trials. Notably, many respondents who had more information about clinical trials were women. We found that most participants were aware that a drug must be tested in a clinical trial before being approved for use. Additionally, they were able to differentiate between clinical trials and other types of research. On the other hand, people who had previously participated in clinical trials had greater knowledge than those who had never done so.

*“A clinical trial is like an experiment to test a drug. You test to see if it is a medicine? Will it help the intended disease? So, once it is accepted, it is sent to people to be used for treatment. But it starts with trials. Without trials, people don’t put medicine into hospitals; it is tested first. So, our children have been tested with the three-hour injection. My son was in the testing of the injection for one and a half year. For example, after finishing the project, that injection dose was distributed to the hospitals for injecting a newborn baby, but they started with experiments.”*

[P8 FGD (female, age 34 years]

*“I know the drug must be tested, because you cannot use something before it has been verified for use.”*

[P1 FGD (female, age 46 years)]

*“I once heard, when I took my son to the project. We were given a lesson and information about the trial project, and we were well-educated. I agreed to take part in the trial, but two of our colleagues denied on the same day and said they were not participating.”*

[P5 FGD (female, age 51 years)]

Furthermore, participants were asked whether they were aware of the advantages, disadvantages, or side effects of taking part in the trial. They were able to explain the advantages and side effects of participating in a clinical trial and that they are always informed by a doctor, who conducts the trial. However, most participants mentioned that clinical trials provided their children with free treatment throughout the trial, as illustrated in the quotes below:

*“There are advantages. For example, my son used to be sick with various diseases, but since I registered him in the project, all the diseases have stopped. In my case, that is a benefit.”*

[P4 FGD (female, age 39 years)]

*“The biggest benefit is getting free health services throughout the project. The services are good and reliable.”*

[P1 FGD (female, age 41 years)]

*“There are side effects. Some suffered side effects, and some did not. There was one whose son got sick right away when he entered the project, but my son didn’t get sick anymore. But the doctor also explained to us about the injections that the child may get convulsions or a high temperature, but he said that it is a normal symptom.”*

[P1 FGD (female, age 46 years)]

*“The drug can be more powerful and affect the person being tested.”*

[P6 FGD (female, age 34 years)]

#### 3.4.2. Attitudes Towards Clinical Trials

In the attitudes section, we wanted to know what factors contribute to a positive or negative attitude towards clinical trials among the Bagamoyo district’s general population. Participants expressed reluctance and concerns about participating in clinical trials. A positive attitude toward clinical trial participation was influenced by the services and care offered by the trials during participation. The majority mentioned these services as an advantage for receiving better treatment when they become sick. This has been illustrated in the quote below:

*“What made me participate were the services provided by the trial. We were told our children would be treated for free. I thought it was better because we pay the clinic money and still don’t get good service. They just prescribe you medicine, and the cost is high. Because of that, it is better to get the project services.”*

[P9 FGD (female, age 32 years]

In addition, the education provided to participants over time has some influence on positive attitudes among community participants. One participant mentioned that

*“Many are interested in the services provided by the project, but we also educate them a lot so that they participate.”*

[P10 IDI (male, age 38 years].

A negative attitude was attributed to misinformation or inadequate information being provided about the trials. Participants mentioned that trials involving blood often require large blood samples, and they sometimes felt uninformed about how their blood would be used. Additionally, some cited peer influence as a source of misinformation, which develops negative attitudes among the community towards clinical trials. Others associated clinical trials with profit-making ventures and were hesitant to participate, feeling that scientists were using them to generate profit.

*“I always get scared because I don’t know what will happen after the project.”*

[P7 FGD (female, age 36 years)]

*“Many are worried due to the wrong information about the projects. Because there is many wrong information, such as sucking blood or drawing a lot of blood from children. So, parents refuse to participate in the projects.”*

[CHW IDI (male, age 56 years)]

*“Many people have been demanding money even before the project has started. From their point of view, they claim that the projects are funded, so scientists get money, while for them, they only volunteer, they don’t get anything, so they are not ready to participate if they are not paid.”*

[CHW IDI (male, age 38 years)]

*“We were told that they suck the blood of children, or draw a lot of blood, then they go to sell abroad”*

[P4 FGD (female, age 39 years)]

*“Many projects involve either donating blood for testing or testing blood. Now, when a citizen hears about donating blood, he is worried. Many say that the blood is sent to be sold abroad because the whites are the ones funding the projects, so they don’t trust the projects at all.”*

[CHW IDI (male, age 56 years)]

## 4. Discussion

The present study aimed to assess the knowledge and attitude of the general population in the Bagamoyo district towards clinical trials. The findings indicated that while the majority of participants held a positive attitude, overall knowledge of clinical trials was low, as supported by data shown in Table 2 and Table 4. Despite participants demonstrating moderate knowledge that drugs are being tested before use and recognising that participation in the study is voluntary, they demonstrated limited knowledge of the meaning of CT and differentiating between a clinical trial and standard care. The majority had a positive attitude, with 89.6% reporting their willingness to participate in CT, 80.7% saying that they would recommend others to participate in the trials, and 90.9% recognising the benefits of CTs.

While other studies have reported similar findings, showing high knowledge and a positive attitude among the general population toward clinical trials [18,34], this study revealed complex public perception toward CTs, with evidence that the majority recognise the benefits of CTs [35]. Despite the high recognition of CTs’ benefits among participants, and the majority (89.6%) expressing their willingness to participate in CTs, interestingly, more than half of participants (53.8%) agreed that CTs harm society. About 31.5% expressed that CT participants are treated as guinea pigs. These contradicting responses suggested that there is a mixture of attitude levels, with a strongly negative perception of CTs in the community. The negative perception aligns with the qualitative findings, in which some participants felt that the blood samples taken during the clinical trial were for sale.

Contrary to other studies, which found that higher levels of education influenced knowledge and attitudes towards clinical trials [23,36], the current study found no significant association between education level or marital status and knowledge or attitude scores. This aligns with findings from a study conducted in India, suggesting that education may not universally predict clinical trial literacy [36].

Interestingly, gender, age, employment status, and income level emerged as significant predictors of knowledge and attitudes. Female participants had greater knowledge and more positive attitudes than male participants. Similar findings were observed in one study performed in China [32], but contrasted with results from Jordan, where men were found to have higher levels of knowledge. In the Bagamoyo context, this may be explained by the higher involvement of women in the paediatric malaria vaccine trials, particularly at the Ifakara Clinical Trial Centre. In African culture, women are the primary caregivers for the children, and that directly affects gender balance in research activities [34].

It was also found that younger and employed individuals had significantly greater knowledge and more positive attitudes [32]. Younger people may have greater health literacy, more exposure to health information, and fewer barriers to participation than older adults. Employed individuals are likely to benefit from access to information and communication technologies, workplace health programmes, and peer networks that facilitate awareness of clinical research [37,38].

Contrary to findings from Jordan and Saudi Arabia, which linked higher income with greater knowledge [23,34], our study observed greater participation and engagement among lower-income individuals. Qualitative data suggested that economic incentives such as free medical care and modest compensation may drive participation among this group, which is similar to a study conducted in Indonesia [39]. This suggests a complex interplay between economic status, perceived benefit, and motivation. While such incentives may raise ethical concerns, particularly regarding the potential for undue inducement, they are typically reviewed and approved by local ethics committees to ensure they are appropriate and ethically sound. Adherence to the Declaration of Helsinki remains essential, guiding ethical review processes to ensure that research addresses the genuine needs of the population without compromising voluntary participation.

### Strengths and Limitations

To the best of our knowledge, this is the first study conducted in Bagamoyo, Tanzania, to assess the knowledge and attitudes of the general population towards clinical trials. The results of this study provide valuable baseline data for researchers conducting CTs who seek to improve recruitment strategies and minimise barriers to enrolling participants in CTs.

However, the study has limitations. The use of random sampling at the household level introduced selection bias, particularly through the overrepresentation of females (74.4% of respondents), as women were more often present at home during the survey than men. This gender imbalance may limit the generalizability of findings, particularly regarding the observed differences between men and women. Furthermore, the current research findings cannot be generalised to other contexts due to differences in social and cultural characteristics across the country.

## 5. Conclusions

This study highlights a disparity between knowledge and attitudes toward clinical trials in the Bagamoyo district. While knowledge remains moderate among the community, the attitude toward participation is generally positive, with a mixture of some negative elements. Younger individuals, females, and employed individuals were found to be more exposed to CT information. These findings underscore the importance of tailored health education interventions, particularly for older adults and the unemployed, who may face barriers to understanding or accessing trial opportunities.

Economic status appears to influence trial engagement, with lower-income individuals more likely to participate, possibly driven by access to free medical care and compensation. While these factors may contribute positively to equitable access and participation, they also underscore the importance of careful ethical oversight to ensure that recruitment remains voluntary, free of undue influence, and appropriate. Ongoing adherence to ethical guidelines and context-specific review by local ethics committees helps safeguard participant welfare and promote fairness in recruitment strategies.

We recommend developing targeted educational interventions to improve clinical trial literacy, increasing access to information for underrepresented and vulnerable populations, ensuring representative sampling in future studies through stratified or oversampling methods, and supporting policy initiatives that fund community outreach and engagement to strengthen public trust and participation in clinical research. It is important to note that these findings are specific to the Bagamoyo district and not necessarily generalizable to all of Tanzania.

## Figures and Tables

**Table 1:** Demographic characteristics of the study population (n = 394) (Researcher, 2024).

	n	%
Gender		
Male	101	25.6
Female	293	74.4
Age		
18–44 years	140	35.5
45–54 years	126	32.0
55 years and above	128	32.5
Level of education		
None + primary Level	266	67.5
Secondary + tertiary level	128	32.5
Villages		
Bago	37	9.4
Kiwangwa	157	39.8
Mazizi	100	25.4
Msata	100	25.4
Employment status		
Employed + self employed	186	47.2
Housewife	104	26.2
Unemployed + Student	104	26.2
Marital status		
Married	297	75.4
Single + Divorced	97	24.6
Income per month (in Tsh)		
less than 50,000	244	61.9
50,000 and above	110	27.9

**Table 2:** Responses of the participants for the knowledge items (n = 394) (Researcher, 2024).

Knowledge About CTs.	Responses	
	Yes (%)	No/Don’t Know (%)
Do you know that the medicines you have been given are tested before use?	228 (60.3)	150 (39.7)
Do you know what clinical trials (CTs) are?	228 (57.9)	116 (42.1)
Do you know the meaning of clinical trials?	186 (47.2)	208 (52.8)
Have you ever heard about the clinical trial centre of the Ifakara health institute in Bagamoyo?	243 (63.8)	138 (36.2)
Do you know the difference between clinical trials and standard care?	174 (46.0)	204 (54.0)
Clinical trials include things like human subjects, testing new drugs, vaccines, medical devices.	222 (58.7)	156 (41.3)
There are benefits to participating in CTs.	231 (58.6)	163 (41.4)
Participating in clinical trials has risks.	102 (25.9)	292 (74.1)
Clinical trial procedures follow a plan known as a protocol.	208 (55.0)	170 (45.0)
Each clinical trial has its own set of rules that determines who may participate.	218 (55.3)	176 (44.7)
Do you know what randomization in clinical trials is?	33 (8.4)	361 (91.6)
Do you know what placebo is?	19 (5.0)	359 (95.0)
Have you ever heard of an ethics review committee for a CT?	55 (14.0)	339 (86.0)
Each clinical trial is conducted by following ethical guidelines for managing CTs?	216 (57.1)	162 (42.9)
Informed consent is a requirement for all clinical trials.	227 (57.6)	167 (42.4)
Can CT be started by the research team without participant consent?	40 (10.2)	354 (89.8)
Participation in research is completely voluntary	265 (67.5)	129 (32.7)
Any participant may leave the CT at any moment.	237 (60.2)	157 (39.8)
A participant’s name or other private information may be disclosed in the published article.	48 (12.2)	346 (87.8)

Legend from Table 2 above: Yes—respondent is aware of clinical trial or has heard about them; No—respondent is not aware of clinical trials; Don’t know—the respondent is unsure or has little trust in their expertise in clinical trials. Note: For analysis purposes, “No and I don’t know” responses were combined to represent a general lack of awareness or uncertainty. Their separate responses are available upon request.

**Table 3:** Factors associated with the Bagamoyo district general population’s knowledge towards CTs. The test applied: binomial logistic regression analysis (n = 394).

Variables	Knowledge Score (%)		Bivariate Analysis		Multivariate Analysis	
	High (n = 205)	Low (n = 189)	Crude OR		Adjusted OR	
Sex			OR (95%CI)	*p*-value	OR (95%CI)	*p*-value
Male	9 (8.9)	92 (91.1)	Ref		Ref	
Female	196 (66.9)	97 (33.1)	20.7 (9.9–42.7)	<0.001	18.1 (8.7, 37.9)	<0.001
Age						
18–44 years	93 (66.4)	47 (33.6)	3.1 (1.9–5.1)	<0.001	3.1 (1.7, 5.7)	<0.001
45–54 years	62 (49.2)	64 (50.8)	1.5 (0.9–2.5)	0.12	1.6 (0.9, 2.8)	0.14
55 years and above	50 (39.1)	78 (60.9)	Ref			
Education level						
none/primary	135 (50.8)	131 (49.2)	1.2 (0.8–1.8)	0.46		
secondary/above	70 (54.7)	58 (45.3)	Ref			
Employment status						
Employed/self-employed	110 (59.1)	76 (40.9)	2.9 (1.7–4.7)	<0.001	2.3 (1.2–4.2)	0.01
Housewife	60 (57.7)	44 (42.3)	2.7 (1.5–4.7)	0.001	1.6 (0.8–3.1)	0.16
Unemployed	35 (33.7)	69 (66.3)	Ref		Ref	
Marital status						
Single + Divorced	54 (55.7)	43 (44.3)	1.2 (0.8–1.9)	0.41		
Married	151 (50.8)	146 (49.2)	Ref			
Income						
less than TZS 50,000	144 (59.0)	100 (41.0)	2.1 (1.4–3.2)	<0.001	2.2 (1.3–3.6)	0.04
TZS 50,000 and above	61 (40.7)	89 (59.3)	Ref		Ref	

Mean score for knowledge (mean ± SD) 7.45 ± 5.56.

**Table 4:** Responses of the participants for attitude towards CTs (n = 394) (Researcher, 2024).

Attitude of Toward CTs	Agree (%)	Disagree/Not Sure (%)
If given adequate information, would you be willing to take part in a CT?	353 (89.6)	41 (10.4)
Clinical trials that are conducted are beneficial to the society.	358 (90.9)	36 (9.1)
Clinical trials conducted harms society	212 (53.8)	182 (46.2)
Clinical trial research is a crucial step in the development of novel medical treatments and products.	333 (84.5)	61 (15.5)
Conducting experiments on humans is essential to the progress of science.	346 (87.8)	48 (12.2)
I would recommend a friend or family member to participate in a clinical trial.	318 (80.7)	76 (19.3)
The way clinical trials are conducted is unethical.	24 (6.1)	370 (93.9)
People participate in clinical trials mainly for financial reasons	162 (41.1)	232 (58.9)
Volunteers must be remunerated while participating in clinical trials	261 (66.2)	133 (33.8)
Privacy is maintained for volunteers involved in clinical trials.	296 (75.1)	98 (24.9)
There are barriers to participating in Clinical trials	225 (57.1)	169 (42.9)
Information on clinical trials can be trusted.	278 (70.6)	116 (29.4)
Patients are forced by doctors to take part in research.	32 (8.1)	362 (91.9)
In clinical research, humans are treated similarly to laboratory animals or “human guinea pigs”.	124 (31.5)	270 (68.5)

**Table 5:** Factors associated with the general population’s attitude towards CTs in the Bagamoyo district. The test applied: binomial logistic regression analysis (n = 394) (Researcher, 2024).

Variables	Attitude Score (%)		Bivariate Analysis		Multivariate Analysis	
	Negative (n = 170)	Positive (n = 224)	Crude OR		Adjusted OR	
Sex			OR (95%CI)	*p*-value	OR (95%CI)	*p*-value
Male	78 (77.2)	23 (22.8)	Ref		Ref	
Female	92 (31.4)	201 (68.9)	7.4 (4.4–12.5)	<0.001	6.2 (3.6–10.8)	<0.001
Age						
18–44 years	45 (32.1)	95 (67.9)	2.6 (1.6–4.3)	<0.001	2.2 (1.2–3.9)	0.007
45–54 years	54 (42.9)	72 (57.1)	1.7 (1.0–2.7)	0.05	1.5 (0.8–2.7)	0.16
55 years and above	71 (55.5)	57 (44.5)	Ref		Ref	
Education level						
none/primary	118 (44.4)	148 (55.6)	0.9 (0.6–1.3)	0.48		
secondary/above	52 (40.6)	76 (59.4)	Ref			
Employment status						
Employed/self-employed	64 (34.7)	122 (65.6)	2.8 (1.7–4.6)	<0.001	2.3 (1.3–4.1)	0.004
Housewife	44 (42.3)	60 (57.7)	2.0 (1.2–3.5)	0.01	1.3 (0.7–2.4)	0.41
Unemployed	62 (59.6)	42 (40.4)	Ref			
Marital status						
Single + Divorced	37 (38.1)	60 (61.9)	1.3 (0.8–2.1)	0.25		
Married	133 (44.8)	164 (55.2)	Ref			
Income						
less than 50,000 TZS	90 (36.9)	154 (63.1)	2.0 (1.3–3.0)	0.001	2.1 (1.3–3.4)	0.003
50,000 TZS and above	80 (53.3)	70 (46.7)	Ref		Ref	

Total mean score of attitude (mean ± SD) = 9.19 ± 3.66.

## Data Availability

The datasets used and/or analysed during the current study are available from the corresponding author upon reasonable request.
